# Endoplasmic reticulum stress induces autophagy and apoptosis while inhibiting proliferation and drug resistance in multiple myeloma through the PI3K/Akt/mTOR signaling pathway

**DOI:** 10.18632/oncotarget.17862

**Published:** 2017-05-15

**Authors:** Yun-Feng Fu, Xiao Liu, Meng Gao, Ya-Nan Zhang, Jing Liu

**Affiliations:** ^1^ The Third Xiangya Hospital of Central South University, Changsha 410013, P.R. China

**Keywords:** multiple myeloma, endoplasmic reticulum stress, PI3K/Akt/mTOR signaling pathway, autophagy, apoptosis

## Abstract

We investigated the effects of endoplasmic reticulum stress (ERS) on autophagy, proliferation, apoptosis, and drug resistance in multiple myeloma (MM). MM patients enrolled in our study (n = 268) were classified into sensitive and resistant groups based on chemotherapy efficacy, and their serum levels of β2-MG, albumin (ALB), lactic dehydrogenase (LDH), Ca^2+^ and hemoglobin were determined. In addition, human MM U266 and MOLP-2/R cells were divided into blank, tunicamycin (TM), TM + insulin-like growth factor-1 (IGF-1), and TM + rapamycin groups, and measured expression of ERS-related, PI3K/Akt/mTOR pathway-related, and autophagy-related mRNA and proteins. Serum levels of β2-MG, LDH and Ca2^+^, and expression of PI3K, Akt, and mTOR were higher in the resistant than sensitive group. Serum levels of ALB and hemoglobin, and expression of glucose-regulated protein 78 (GRP78), GRP94, microtubule associated protein 1 light chain 3 (LC3), and Beclin1, were lower in the resistant than sensitive group. In U266 cells treated with TM and IGF-1 or rapamycin, ERS promoted autophagy and apoptosis while inhibiting proliferation through inhibition of PI3K/Akt/mTOR signaling. ERS also reversed drug resistance in MOLP-2/R cells via the PI3K/Akt/mTOR signaling pathway. These data suggest that ERS activation could be exploited for therapeutic benefits in the treatment of MM.

## INTRODUCTION

Multiple myeloma (MM) is a B-cell malignancy characterized by the amplification of clonal plasma blasts or plasma cells (PC) in the bone marrow (BM) through pathways activated by tyrosine kinase [1]. MM results from vicious PC gathering in the BM and leads to lytic bone lesions [2]. MM accounts for 10% of hematologic malignant tumors, and its clinical characteristics include bone pain, renal failure, susceptibility to infections, anemia, and hypercalcaemia [2]. In the United States, MM is slightly more prevalent in men than in women, and the average age of patients at diagnosis is 65 years [3]. Bortezomib (BTZ), a proteasome inhibitor, was initially reported as an inhibitor of the NF-κB pathway, which promotes MM pathogenesis [4]. Recently, proteasome inhibition in MM cells was shown to cause an accumulation of unfolded proteins in the endoplasmic reticulum (ER) referred to as ER stress (ERS), and to induce apoptosis [4].

The ER, the site where secretory proteins are synthesized and folded, is necessary for most cellular activities [5] and perturbations to ER homeostasis leads to ERS. However, the ER can sense the stress and reacts to it by attenuating translation, upregulating genes that code for ER chaperones and other relevant proteins, and degrading unfolded proteins [6]. Autophagy, as a lysosomal degradation pathway, is necessary for the survival, development, homeostasis and differentiation of cells, and it plays an adaptive role to protect organisms against diverse pathologies [7]. The unfolding protein response (UPR) and autophagy are simultaneously involved in tumorigenesis, chemoresistance of malignancies, and neurodegeneration [8]. The phosphatidylinositol 3-kinase (PI3K)/Akt/mammalian target of rapamycin (mTOR) signaling pathway axis promotes cell growth, survival, and proliferation [9]. Various types of human cancers exhibit aberrancies in the PI3K/Akt/mTOR signaling pathway; therefore, this pathway might be a useful clinical target for cancer treatment [10]. Here, we investigated the role of ERS in MM cell proliferation and drug resistance.

## RESULTS

### The drug-resistant group showed higher serum levels of β2-MG, LDH and Ca^2+^ but lower levels of ALB and hemoglobin

The serum levels of β2-MG, LDH and Ca^2+^ were increased and those of ALB and hemoglobin were decreasedin the resistant group compared with the sensitive group (all *P* < 0.05). There was no association between the serum levels of those indexes with the disease stage, age, and gender of patients (all *P* > 0.05) (Table 1).

**Table 1:** Comparisons of serum indexes between the sensitive and resistant groups

	Sensitive group (n = 130)	Resistant group (n = 138)	*P*
Age	56.65 ± 11.21	54.10 ± 13.18	0.090
Gender			
Male	76	90	0.255
Female	54	48	
Tumor staging			
Stage I	14	14*	0.573
Stage II	34	29*	
Stage III	82	95*	
β2-MG (mg/L)	2.67 ± 0.87	5.76 ± 1.34*	< 0.001
ALB (g/L)	43.21 ± 5.34	34.82 ± 3.21*	< 0.001
LDH (U/L)	176.38 ± 15.43	307.03± 43.10*	< 0.001
Ca2^+^ (mmol/L)	2.13 ± 0.12	2.97 ± 0.24*	< 0.001
hemoglobin (g/L)	113.10 ± 11.74	94.01 ± 9.12*	< 0.001

Note: ALB, albumin; LDH, lactic dehydrogenase; *, *P* < 0.05 compared with the sensitive group.

### Comparisons of mRNA and protein expressions of GRP78, GRP94, PI3K, Akt, mTOR, LC3 and Beclin1 between the sensitive and resistant groups

**Figure 1:**
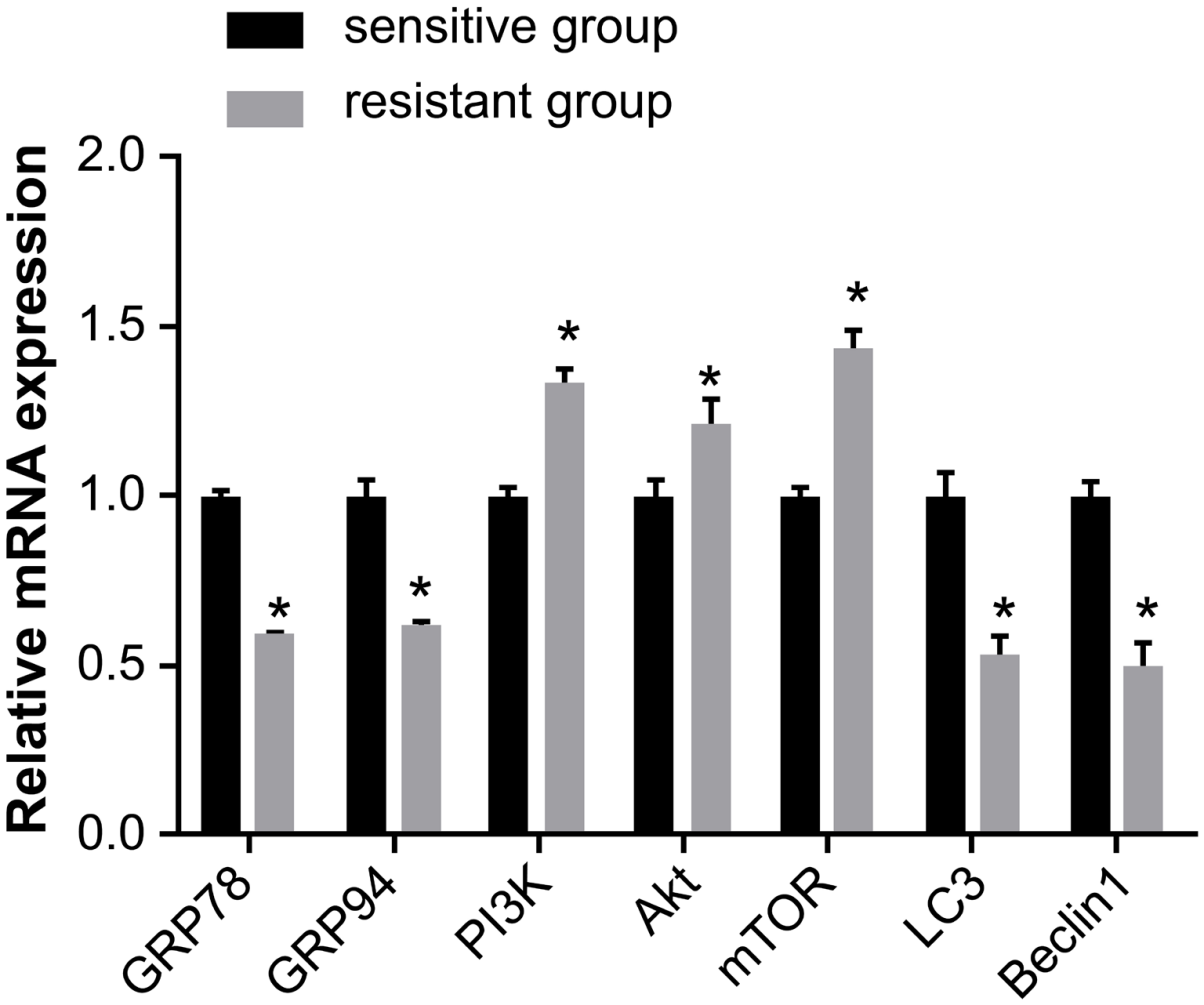
Comparison of the mRNA expression of GRP78, GRP94, PI3K, Akt, mTOR, LC3 and Beclin1 between the sensitive and resistant groups Note: *, *P* < 0.05 compared to the sensitive group; GRP, glucose-regulated protein 78; PI3K, phosphatidylinositol 3-kinase; mTOR, mammalian target of rapamycin; LC3, microtubule associated protein 1 light chain 3.

According to qRT-PCR, the mRNA expression of GRP78, GRP94, LC3, and Beclin1 was lower in the resistant group than in the sensitive group, while the mRNA expression of PI3K, Akt, and mTOR was higher (all *P* < 0.05) (Figure 1).

**Figure 2:**
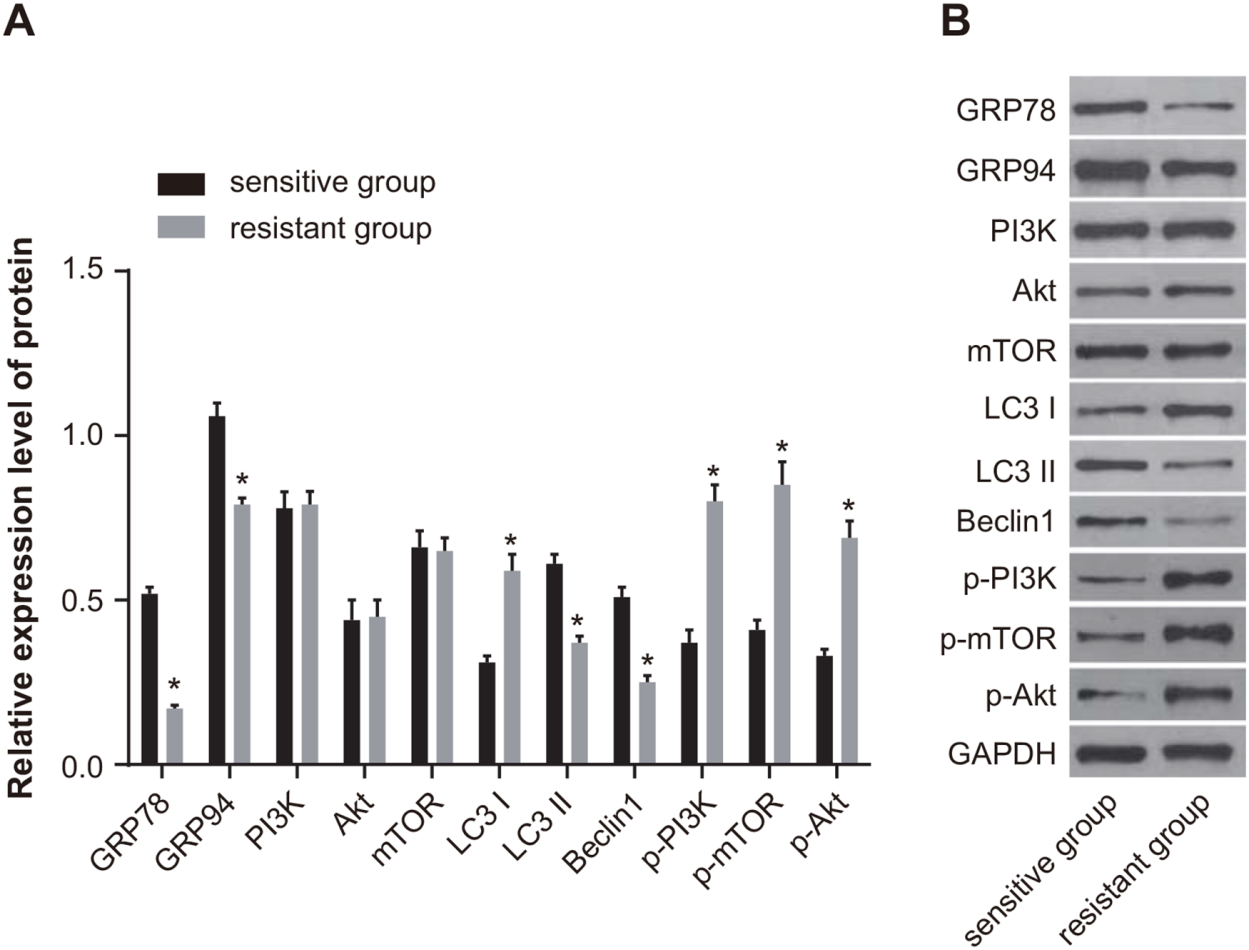
Comparison of the protein expression of GRP78, GRP94, PI3K, Akt, mTOR, LC3 and Beclin1 between the sensitive and resistant groups **(A)** Histogram showing protein expressions by Western blotting. **(B)** Protein bands showing relative protein expression. Note: *, *P* < 0.05 compared to the sensitive group; GRP, glucose-regulated protein 78; PI3K, phosphatidylinositol 3-kinase; mTOR, mammalian target of rapamycin; LC3, microtubule associated protein 1 light chain 3.

Western blotting showed that the expression of GRP78, GRP94, LC3 II, and Beclin1 was downregulated in the resistant group compared with the sensitive group. On the other hand, the expression of p-PI3K, p-Akt, p-mTOR and LC3 I (all *P* < 0.05) was upregulated in the resistant group. There was no difference in the protein expression of PI3K, Akt, and mTOR between the sensitive and resistant groups (all *P* > 0.05) (Figure 2).

### Expression of ERS-related mRNA and PI3K/Akt/mTOR signaling pathway-related mRNA in U266 cells

**Figure 3:**
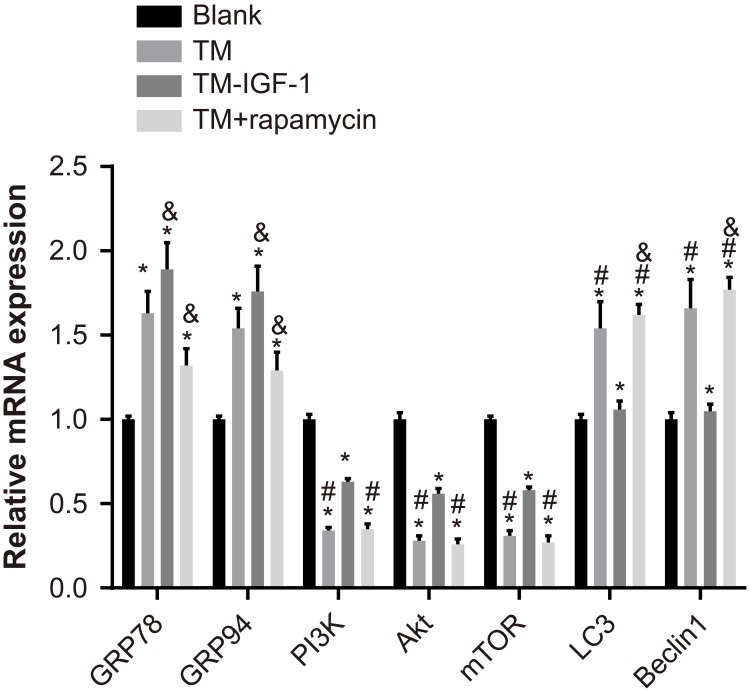
Expression of ERS-related mRNA and PI3K/Akt/mTOR signaling pathway-related mRNA in U266 cells in the blank, TM, TM + IGF-1, and TM + rapamycin groups Note: *, *P* < 0.05 compared to the blank group; #, *P* < 0.05 compared with the blank and TM + IGF-1 groups; ^&^, *P* < 0.05 compared with the TM group; ERS, endoplasmic reticulum stress; PI3K, phosphatidylinositol 3-kinase; mTOR, mammalian target of rapamycin; TM, tunicamycin; IGF-1, insulin-like growth factor-1.

The mRNA expression of GRP78 and GRP94 was elevated in the TM, TM + IGF-1, and TM + rapamycin groups compared with the blank group, but the mRNA expression of PI3K, Akt, and mTOR was reduced (all *P* < 0.05). Compared with the blank and TM + IGF-1 groups, the TM and TM + rapamycin groups showed increased mRNA expression of LC3 and Beclin1 and decreased mRNA expression of PI3K, Akt, and mTOR (all *P* < 0.05). There was no difference in the mRNA expression of LC3 and Beclin1 between the blank and TM + IGF-1 groups (all *P* > 0.05). The mRNA expression of GRP78 and GRP94 was increased in the TM + IGF-1 group but decreased in the TM + rapamycin group compared with the TM group (all *P* < 0.05). The mRNA expression of LC3 and Beclin1 was higher in the TM + rapamycin group than in the TM group (all *P*> 0.05) (Figure 3).

### Expression of ERS-related proteins and PI3K/Akt/mTOR signaling pathway-related proteins in U266 cells

**Figure 4:**
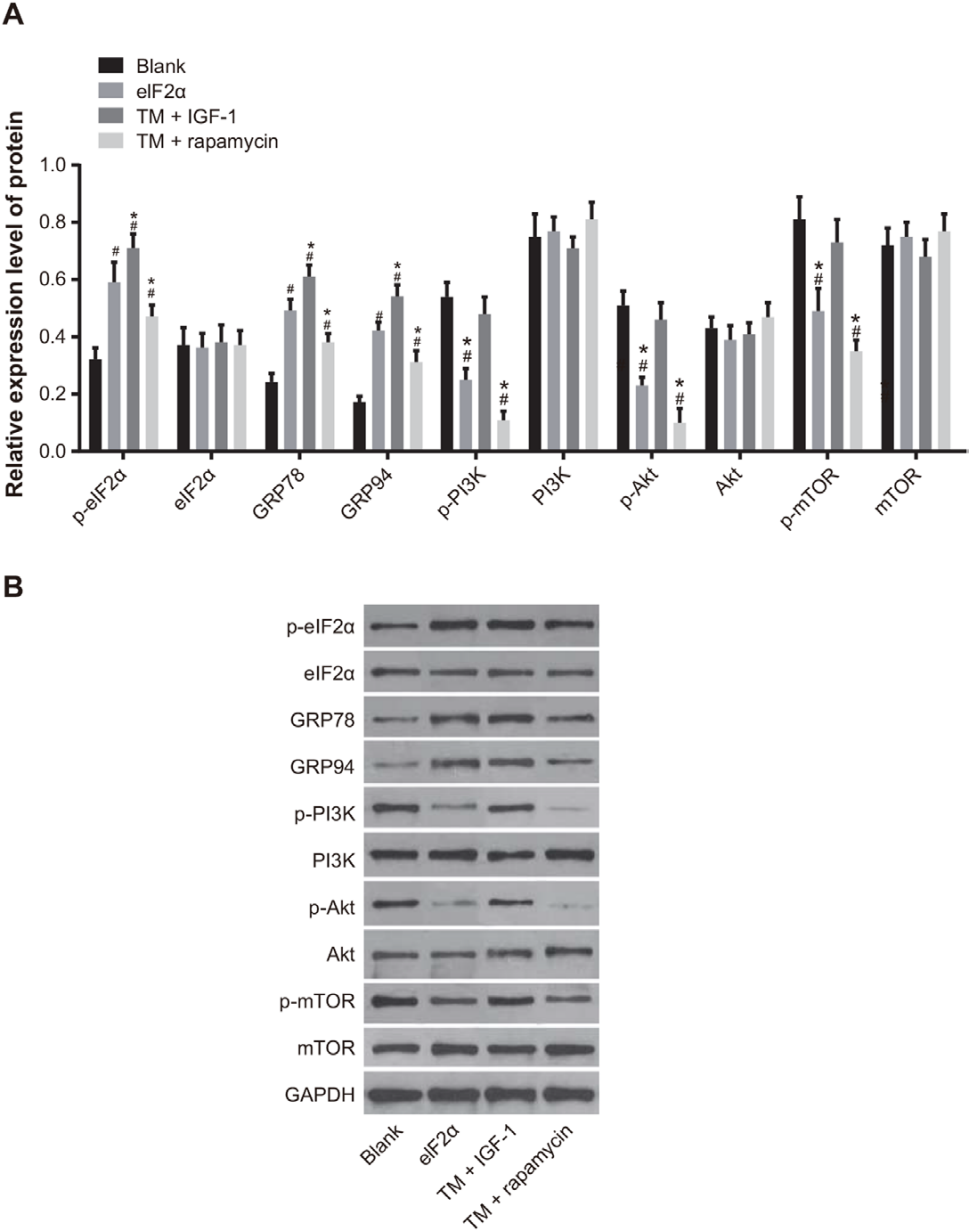
Expression of ERS-related proteins and PI3K/Akt/mTOR signaling pathway-related proteins in U266 cells in the blank, TM, TM + rapamycin and TM + IGF-1 groups measured by Western blotting **(A)** Histogram showing the expressions of ERS-related proteins and PI3K/Akt/mTOR signaling pathway-related proteins in U266 cells among the four groups. **(B)** Protein bands showing the expressions of ERS-related proteins and PI3K/Akt/mTOR signaling pathway-related proteins in U266 cells in all four groups. Note: #, *P* < 0.05 compared with the blank group; *, *P* < 0.05 compared with the TM group; ERS, endoplasmic reticulum stress; PI3K, phosphatidylinositol 3-kinase; mTOR, mammalian target of rapamycin; TM, tunicamycin; IGF-1, insulin-like growth factor-1.

The protein expression of p-eIF2α, GRP78, and GRP94 was increased in the TM, TM + IGF-1, and TM + rapamycin groups compared with the blank group (all *P* < 0.05). The protein expression of p-eIF2α, GRP78, and GRP94 was upregulated in the TM + IGF-1 group (all *P* < 0.05), but downregulated in the TM + rapamycin group compared with the TM group (all *P* < 0.05). The protein expression of eIF2α in each group had no obvious changes (all *P* > 0.05). The protein expression of p-PI3K, p-Akt, and p-mTOR was decreased in the TM and TM + rapamycin groups compared with the blank group (all *P* < 0.05). There was no difference between the protein expression of p-PI3K, p-Akt, and p-mTOR in the TM + IGF-1 and the blank groups (all *P* > 0.05). The protein expression of p-PI3K, p-Akt, and p-mTOR was increased in the TM + IGF-1 group (all *P* < 0.05), but was decreased in the TM + rapamycin group (all *P* < 0.05). There was no difference in the protein expression of PI3K, Akt, and mTOR among the blank, TM, TM + IGF-1, and TM + rapamycin groups (all *P*> 0.05) (Figure 4A, 4B).

### ES promotes autophagy and apoptosis in U266 cells by inhibiting the PI3K/Akt/mTOR signaling pathway

**Figure 5:**
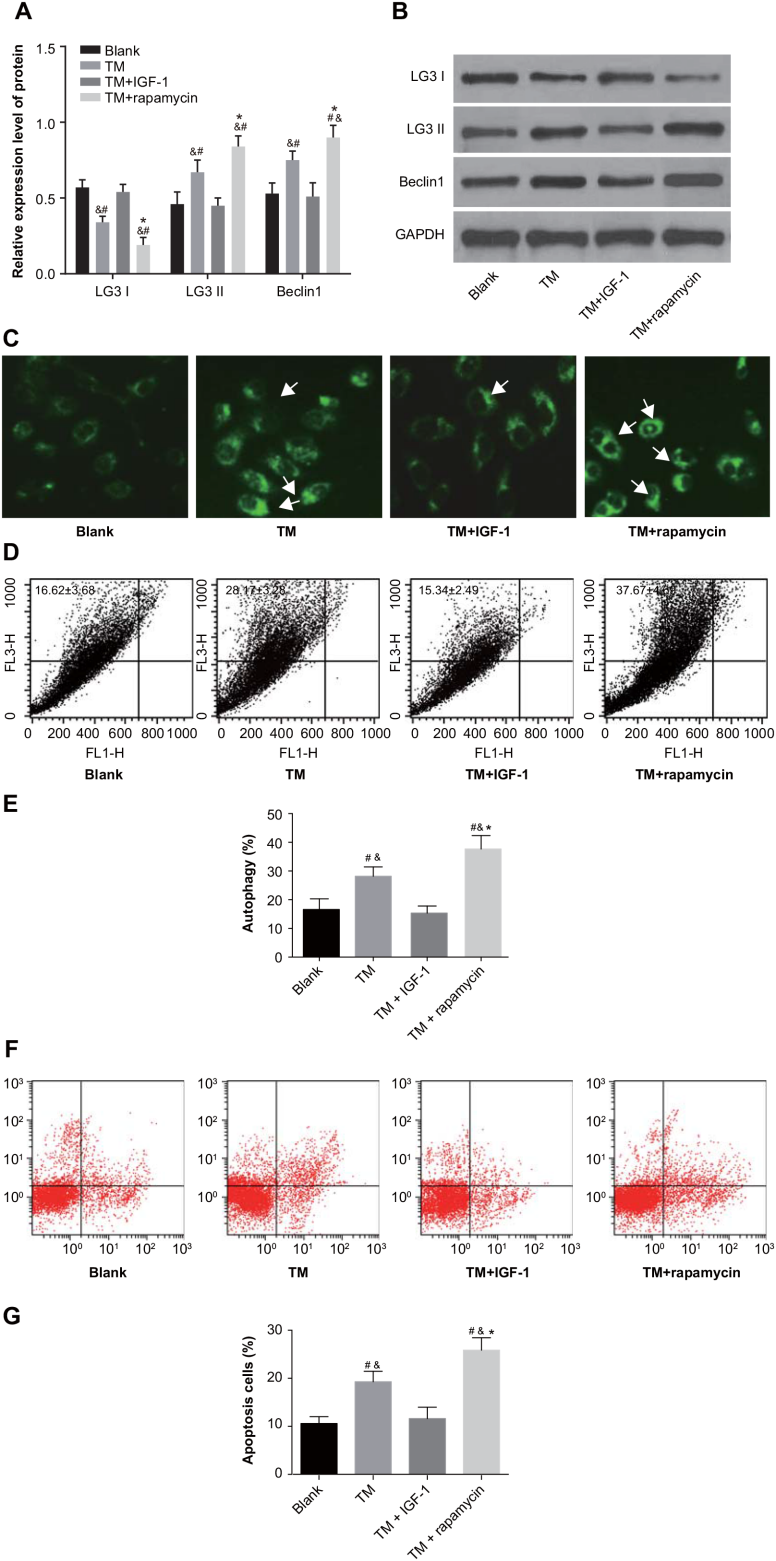
ERS promotes autophagy and apoptosis in U266 cells through inhibition of the PI3K/Akt/mTOR signaling pathway in the blank, TM, TM + rapamycin and TM + IGF-1 groups **(A)** Histogram showing the comparisons of the expressions of LC3 I, LC3 II and Beclin1 proteins in U266 cells among the four groups. **(B)** Protein bands showing the expressions of LC3 I, LC3 II and Beclin1 proteins in U266 cells in all four groups. **(C)** Detection of autophagy in U266 cells by MDC staining. **(D)** Detection of autophagy rate in U266 cells by FL3/FL1. **(E)** Histogram showing the comparison of the autophagy rate of U266 cells among the four groups. **(F)** Detection of apoptosis in U266 cells by Annexin V-FITC/PI. **(G)** Histogram showing the comparison of the apoptosis of U266 cells among the four groups. Note: #, *P* < 0.05 compared with the blank group; *, *P* < 0.05 compared with the TM group; &, *P* < 0.05 compared with the TM + IGF-1 group; ERS, endoplasmic reticulum stress; PI3K, phosphatidylinositol 3-kinase; mTOR, mammalian target of rapamycin; TM, tunicamycin; IGF-1, insulin-like growth factor-1; MDC, monodansylcadaverine; FL3, the third fluorescence channel; FL1, the first fluorescence channel; LC3 I, microtubule associated protein 1 light chain 3 I; LC3 II, microtubule associated protein 1 light chain 3 II; Annexin V-FITC/PI, Annexin V-fluorescein isothiocyanate/propidium iodide.

MDC staining showed high fluorescence intensity in the TM and TM + rapamycin groups with aggregated fluorescent particles visible in the cytoplasm. On the other hand, fluorescence intensity in the blank and TM + IGF-1 groups was relatively weak (Figure 5C). Western blotting showed that compared with the blank and TM + IGF-1 groups, the protein expression of LC3 II and Beclin1 was increased (all *P* < 0.05) while that of LC3 I was decreased (all *P* < 0.05) in the TM and TM + rapamycin groups. There was no difference in the protein expression of LC3 I, LC3 II and Beclin1 between the blank and TM + IGF-1 groups (all *P* > 0.05). Compared with the TM group, the protein expression of LC3 II and Beclin1 was increased while the protein expression of LC3 I was decreased in the TM + rapamycin group (all *P* < 0.05) (Figure 5A, 5B). The rates of autophagy and apoptosis in U266 cells measured was higher in the TM and TM + rapamycin groups compared with the blank and TM + IGF-1 groups, as measured by flow cytometry (all *P* < 0.05). However, there was no difference in the rates of autophagy and apoptosis between the blank and TM + IGF-1 groups (all *P* > 0.05). The rates of autophagy and apoptosis were elevated in the TM + rapamycin group compared with the TM group (all *P* < 0.05) (Figure 5D–5G).

### ERS reduces the proliferation of U266 cells by inhibiting PI3K/Akt/mTOR signaling pathway

**Figure 6:**
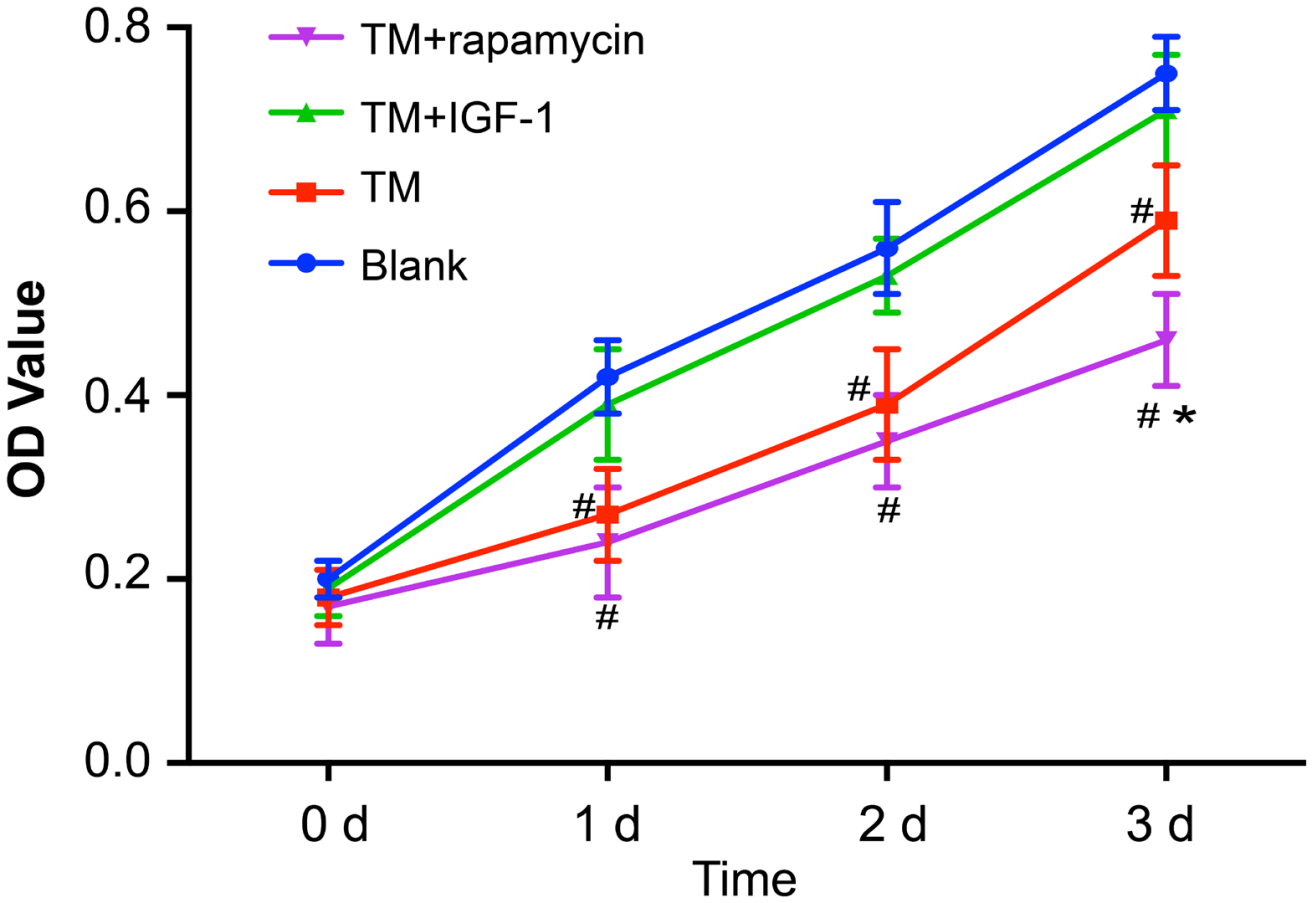
Proliferation of U266 cells in the blank, TM, TM + rapamycin and TM + IGF-1 groups detected by CKK-8 assay at 0 d, 1 d, 2 d and 3 d Note: ^#^, *P* < 0.05 compared with the blank group; *, *P* < 0.05 compared with the TM group; TM, tunicamycin; IGF-1, insulin-like growth factor-1; CCK-8, cell count kit-8.

The proliferation of U266 cells at 0 d, 1 d, 2 d and 3 d detected by CCK-8 showed that there were no differences in OD values among the blank, TM, TM + IGF-1, and TM + rapamycin groups at 0 d (all *P* > 0.05). Compared with the blank group, the OD values in the TM and TM + rapamycin groups were decreased at 1 d, 2 d, and 3 d (all *P* < 0.05), but those in the TM + IGF-1 group had no change (all *P* > 0.05). Compared with the TM group, the OD values in the TM + rapamycin group were decreased at 3 d (all *P* < 0.05), but there were no changes at 1 d and 2 d (all *P* > 0.05) (Figure 6).

### ERS reverses drug resistance of MOLP-2/R cells by inhibiting the PI3K/Akt/mTOR signaling pathway

**Figure 7:**
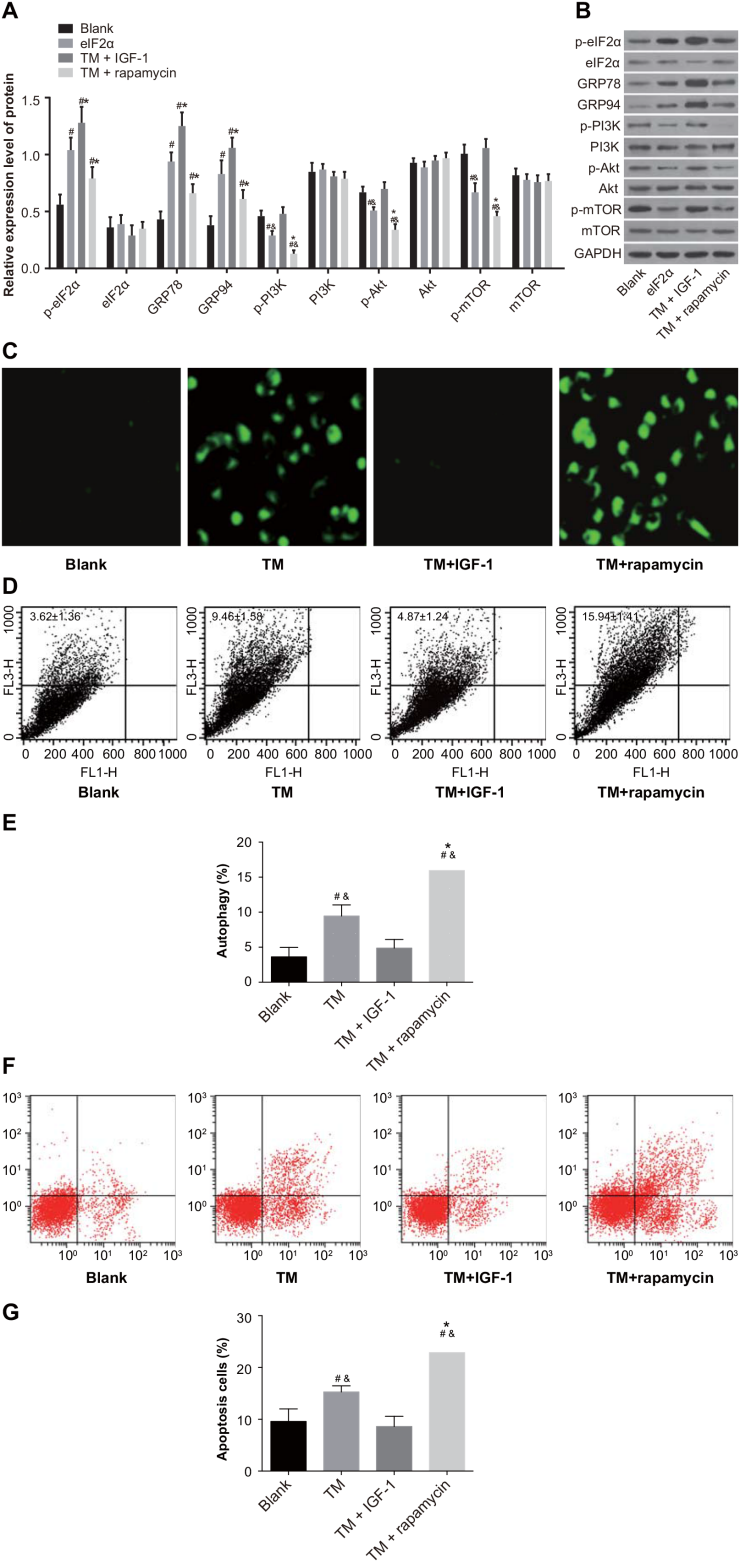
ERS reverses the drug resistance of MOLP-2/R cells by inhibiting PI3K/Akt/mTOR signaling pathway in the blank, TM, TM + rapamycin and TM + IGF-1 groups **(A)** Histogram showing the comparisons of the expressions of ERS-related proteins and PI3K/Akt/mTOR signaling pathway-related proteins in MOLP-2/R cells among the four groups. **(B)** Protein bands showing the expressions of ERS-related proteins and PI3K/Akt/mTOR signaling pathway-related proteins in MOLP-2/R cells in all four groups. **(C)** Detection of autophagy of MOLP-2/R cells by MDC staining. **(D)** Measurement of autophagy rate of MOLP-2/R cells by FL3/FL1. **(E)** Histogram showing the comparison of the autophagy rate of MOLP-2/R cells among the four groups. **(F)** Detection of apoptosis in MOLP-2/R cells by Annexin V-FITC/PI. **(G)** Histogram showing the comparison of the apoptosis rate of MOLP-2/R cells among the four groups. Note: ^#^, *P* < 0.05 compared with the blank group; *, *P* < 0.05 compared with the TM group; ERS, endoplasmic reticulum stress; PI3K, phosphatidylinositol 3-kinase; mTOR, mammalian target of rapamycin; TM, tunicamycin; IGF-1, insulin-like growth factor-1; MDC, monodansylcadaverine; FL3, the third fluorescence channel; FL1, the first fluorescence channel; Annexin V-FITC/PI, Annexin V-fluorescein isothiocyanate/propidium iodide.

**Figure 8:**
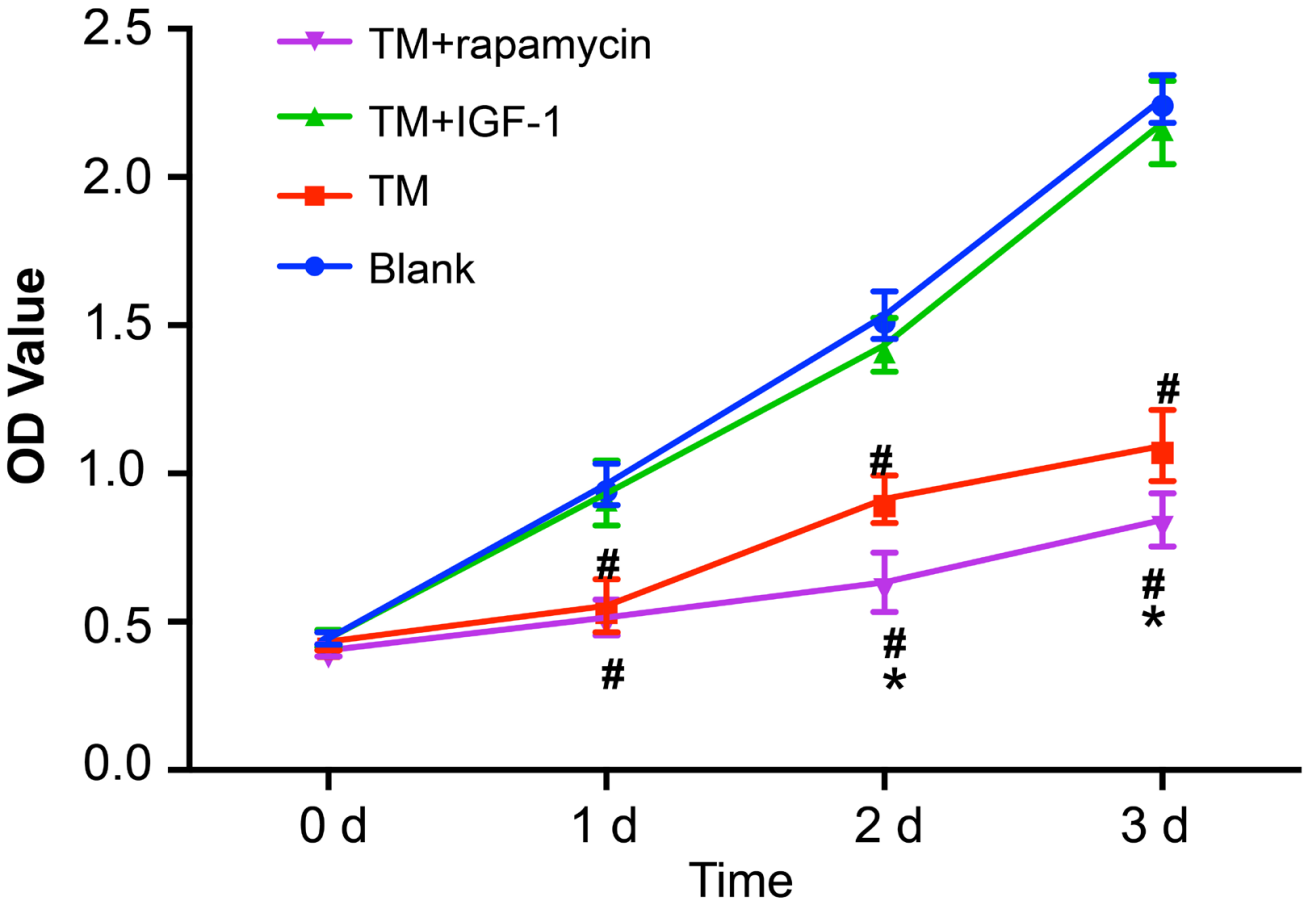
Detection of proliferation of MOLP-2/R cells in the blank, TM, TM + rapamycin and TM + IGF-1 groups by CCK-8 assay Note: TM, tunicamycin; IGF-1, insulin-like growth factor-1; CCK-8, cell count kit-8.

Compared with the blank group, the protein expression of p-eIF2α, GRP78, and GRP94 was upregulated in the TM, TM + IGF-1 and TM + rapamycin groups (all *P* < 0.05). Compared with the TM group, the protein expression of p-eIF2α, GRP78, and GRP94 was also increased in the TM + IGF-1 group (all *P* < 0.05) while that in the TM + rapamycin group was decreased (all *P* < 0.05). There was no difference in the protein expression of eIF2α among all four groups (all *P* > 0.05) (Figure 7A, 7B). The protein expression of p-PI3K, p-Akt, and p-mTOR was decreased in the TM and TM + rapamycin groups compared with the blank and TM + IGF-1 groups (Figure 7A, 7B), but the rates of autophagy and apoptosis were increased (*P* < 0.05) (Figure 7C–7G). There was no difference in the protein expression of p-PI3K, p-Akt, and p-mTOR nor in the rates of autophagy and apoptosis between the TM + IGF-1 group and the blank group (all *P* > 0.05). Compared with the TM group, the protein expression of p-PI3K, p-Akt, and p-mTOR were decreased in the TM + rapamycin group, but the rates of autophagy and apoptosis were increased (all *P* < 0.05). There was no difference in the protein expression of PI3K, Akt, and mTOR among the blank, TM, TM + IGF-1 and TM + rapamycin groups (all *P* > 0.05). CCK-8 assays showed that there were no differences in the OD values among the blank, TM, TM + IGF-1, and TM + rapamycin groups at 0 d (all *P* > 0.05) (Figure 8). Compared with the blank and TM + IGF-1 groups, the OD values in the TM and TM + rapamycin groups were decreased at 1 d, 2 d, and 3 d (all *P* < 0.05), but there were no differences in the OD values between the blank and TM + IGF-1 groups at all time points (all *P* > 0.05). Compared with the TM group, the OD values at 2 d and 3 d were decreased in the TM + rapamycin group (all *P* < 0.05).

## DISCUSSION

In this study, we explored the effect of ERS on the autophagy, apoptosis, growth and drug resistance of MM cell lines. Our results showed that by inhibiting the PI3K/Akt/mTOR signaling pathway, ERS induced autophagy and apoptosis while suppressing proliferation and reversing drug resistance in MM cell lines.

The expression of ERS-related proteins increased in the TM, TM + IGF-1, and TM + rapamycin groups, implying that tunicamycin induced ERS. Accumulation of improperly folded proteins in the ER is attributed to extracellular stimuli and intracellular homeostatic changes that can lead to ERS [11, 12]. Tunicamycin is commonly used to trigger ERS [13–15]. ERS can induce the unfolded protein response (UPR), a compensatory mechanism that counteracts ERS by restoring the homeostasis of protein folding [16]. P-eIF2a, GRP78, and GRP94 expression are hallmarks of ERS and UPR [17, 18]. As expected, tunicamycin elevated the expression of these proteins in our experiments.

The protein expression of p-PI3K, p-Akt, and p-mTOR was much higher in the TM + IGF-1 group than the TM group, indicating that IGF-1 could activate the PI3K/Akt/mTOR signaling pathway. Abnormal activation of the PI3K/Akt/mTOR pathway has been found in various cancers and has been suggested to stimulate proliferation and drug resistance [19]. IGF-1 is a peptide that promotes mitosis and deters apoptosis. High concentrations of IGF-1 in plasma are positively correlated with breast cancer [20]. IGF-1 stimulates protein synthesis by upregulating phosphorylation of PI3K, Akt, and mTOR [21]. We observed lower protein expression of p-PI3K, p-Akt, and p-mTOR in the TM + rapamycin group compared with the TM group, which indicated that rapamycin could inhibit the PI3K/Akt/mTOR signaling pathway, in agreement with a previous study [22]. Rapamycin plays an important role in extending life span by inhibiting the mTOR pathway, which is associated with growth and metabolism [23, 24]. The Protein expression of LC3 II and Beclin1 was higher in the TM + rapamycin group compared with the TM group, which suggested that downregulation of the PI3K/Akt/mTOR signaling pathway correlates positively correlated with cell autophagy. Indeed, LC3 II and Beclin1 genes are associated with autophagy and lower mRNA and protein expression of LC3 II and Beclin1 correlate with tumor progression [25, 26]. Previous studies showed that inhibition of the PI3K/Akt/mTOR signaling pathway enhanced autophagy [27, 28], which is consistent with the higher autophagy and apoptosis rates we observed in the TM and TM + rapamycin groups compared with the blank and TM + IGF-1 groups. A previous study demonstrated that ERS induced cancer cell death by stimulating autophagy [29]. Therefore, ERS can promote autophagy and apoptosis by suppressing the PI3K/Akt/mTOR signaling pathway.

Since activation of the PI3K/Akt/mTOR signaling pathway has been shown to positively correlate with cellular proliferation [30, 31], we hypothesized that the inactivation of this pathway could suppress cellular proliferation. The results of our CCK-8 assays demonstrated that ERS can also suppresses cancer cell proliferation. MM, featured by malignant proliferation of plasma cells in B-lymphocytes, used to be regarded as an incurable disease with an extremely high recurrence rate; however, recent developments have lead to an improved prognosis for MM patients [32]. The introduction of bortezomib (BZ), a proteasome inhibitor, was a breakthrough in the treatment of MM due to its ability to trigger ERS [33]. Furthermore, SCIO-469 treatment can also inhibit MM cell proliferation and adhesion to alleviate osteolytic activation in MM [34]. Several previous studies proposed that ERS played a pivotal role in suppressing cellular proliferation [35–37].

Finally, we found here that ERS could reverse drug resistance in MOLP-2/R cells. Earlier studies found that inhibition of the PI3K/Akt/mTOR signaling pathway reversed resistance in several types of cancer [19, 38, 39]. Autophagy is a conserved catabolic process where long-lived cellular proteins and damaged organelles are engulfed and degraded for recycling to maintain homeostasis [8]. Activation of autophagy in MM cells may reduce drug resistance in myeloma [40]. Taken together, our data suggest that ERS reverses drug resistance in MM cells by stimulating autophagy and inhibiting the PI3K/Akt/mTOR signaling pathway.

In summary, our study here showed that ERS suppressed the PI3K/Akt/mTOR signaling pathway, thereby promoting autophagy and apoptosis while inhibiting proliferation and reversing drug resistance in MM cells. While further research is needed to fully elucidate the mechanisms underlying the effects of ERS on MM cells that we report here, our data suggest that ERS activation in combination with other therapies might yield clinical benefits to MM patients.

## MATERIALS AND METHODS

### Ethics statement

This study was approved by the Ethics Committee of the Third Xiangya Hospital of Central South University. Written informed consent was obtained from all study subjects.

### Study subjects

From April 2010 to August 2014, 268 patients (166 males, 102 females) diagnosed with MM were enrolled in this study. Their ages ranged from 22 to 85 years, with a mean age of 55 years. According to the Durie-Salmon staging system, 48 patients had stage I, 94 had stage II, and 126 had stage III MM. Based on the Southwest Oncology Group (SWOG), there were 62 patients with stage I MM, 98 patients with stage II MM, and 108 patients with stage III ∼ IV MM. Tissue specimens were collected from each patient and preserved by freezing in liquid-nitrogen and stored at -80°C.

### Therapeutic evaluation

All patients underwent VAMD chemotherapy (VCR 0.5 mg d_1-4_, THP 10 mg d_1-4_, melphalan 6 mg d_1-4_ [twice a day], and DXM 20 mg d_1-4_). The therapeutic effect was observed after 2 to 3 courses of treatment. According to the European Group for Blood and Marrow Transplantation (EMBT) criteria, the response can be classified as complete remission (CR), near complete remission (nCR), partial response (PR), minimal response (MR), no change (NC), and progressive disease (PD). In the present study, the patients with CR, nCR and PR were assigned to the sensitive group, while those with MR, NC and PD were assigned to the resistant group.

### Serum index detection

Serum levels of β2-MG, albumin (ALB), lactic dehydrogenase (LDH) and Ca^2+^ were determined using an automatic biochemical analyzer while the level of hemoglobin was determined by using an automatic blood analyzer. Differences in the values measured for the sensitive and drug-resistant groups were further analyzed.

### Quantitative real-time polymerase chain reaction (qRT-PCR)

Total RNA was extracted using RNA extraction kit (10296010; Invitrogen Chinese Inc., Shanghai, China). After testing for purity and completeness, the RNA was reverse transcribed into cDNA using the PrimeScript™ RT kit (RR014A; Takara Biomedical Technology (Beijing) Co., Ltd., Beijing, China). Reverse transcription (10 μL of system) was conducted in triplicate at 37°C for 15 min, and the reverse transcriptase was inactivated at 85°C for 5 s. The primers of glucose-regulated protein 78 (GRP78), GRP94, PI3K, Akt, mTOR, microtubule associated protein 1 light chain 3 (LC3), Beclin1 and glyceraldehyde-3-phosphate dehydrogenase (GAPDH) were synthesized by Takara Holdings Inc. (Kyoto, Japan). Their sequences are shown in Table 2. qRT-PCR was performed using a quantitative PCR kit according to the manufacturer’s protocol (KR011A1; TIANGEN Biotechnology Co. Ltd, Beijing, China). The reaction conditions were as follows: initial denaturation at 95°C for 5 min, 30 cycles at 95°C for 40 s, 57°C for 40 s, 72°C for 40 s, and finally extension at 72°C for 10 min and at 4°C for 5 min. The qRT-PCR system included 10 μL of SYBR Premix Ex TaqTM II, 0.4 μL of PCR Forward Primer (10 μM), 0.4 μL of PCR Reverse Primer (10 μM), 2 μL of DNA template and 7.2 μL of sterilized distilled water. Using GAPDH as internal control, the relative expressions of GRP78, GRP94, PI3K, Akt, mTOR, LC3 and Beclin1 were calculated using the 2-^ΔΔCt^ method. The CT values of GRP78, GRP94, PI3K, Akt, mTOR, LC3, Beclin1, and GAPDH were measured with LightCycler. The qRT-PCR experiment was performed on the MM cells (U266 and MOLP-2) in the same way.

**Table 2:** Sequences of the qRT-PCR primers

Gene	Sequence (5’-3’)
GRP78	F: TCATCGGACGCACTTGGAA
	R: AACCACCTTGAATGGCAAGAA
GRP94	F: CAGTTTTGGATGCTTGCTGTGG
	R: CAGCTGTAGATTCCTTTGC
PI3K	F: GGCTTGGACCTGCGAAT
	R: TTGTTGAAGGCTGCTGTGGC
Akt	F: AGTGACTCCTCCACGACTGAG
	R: GGTGACTGTGTGAGCGACTTC
mTOR	F: TGATGGTGAGTGAAGAGCTGATTCGGGTAG
	R: TTGGTGGACAGAGGGATGACAGCGTATCTC
LC3	F: ATCTCGAGATGCCGTGGGAGAAGACC
	R: CGAATTCTTACACTGACAATTTCATCCCG
Beclin1	F: TAGGATCCATGGAAGGGTCTAAGAC
	R: GCGAAGCTTTCATTGTTATAAAAT
GAPDH	F: CCACCCATGGCAAATTCCATGGCA
	R: TCTAGACGGCAGGTCAGGTCCAC

Note: GRP, glucose-regulated protein 78; PI3K, phosphatidylinositol 3-kinase; mTOR, mammalian target of rapamycin; LC3, microtubule associated protein 1 light chain 3; GAPDH, glyceraldehyde-3-phosphate dehydrogenase; F, forward; R, reverse.

### Western blotting

After 24 h, the total protein was extracted and the protein concentration was determined using bovine carbonic anhydrase (BCA) kit according to the manufacturer’s instructions (AR0145, Wuhan Boster Biological Engineering Co., Ltd., Wuhan, China). The extracted protein was added to the loading buffer and boiled at 95°C for 5 min, with 30 μg of loading buffer in each well. The proteins were separated by 10% polyacrylamide gel electrophoresis (electrophoresis voltage was transferred from 80 v to 120 v), transferred to the polyvinylidene vinyl fluoride (PVDF) membrane (voltage: 100 mv; time: 45-70 min) and blocked by 5% bovine serum albumin (BSA) for 1 h at the room temperature. The membrane was subsequently incubated with primary antibodies at 4°C overnight, including phosphorylated-eukaryotic translational initiation factor 2 (phospho-eIF2α) (Ser51) (1 : 1000, 9721, CST, USA), eukaryotic translational initiation factor 2 (eIF2α) (1 : 1000, 9722, CST, USA), GRP78 (1 : 500, sc-1050, SANTA CRUZ, USA), GRP94 (1 : 500, sc-11402, SANTA CRUZ, USA), PI3K (1 : 1000, 4255, CST, USA), phosphorylated-PI3K (phospho-PI3K) (Ser473) (1 : 1000, 4228, CST, USA), AKT (1 : 1000, 9272, CST, USA), phosphorylated-AKT (phospho-AKT) (Ser496) (1 : 1000, 9271, CST, USA), mTOR (1 : 1000, 2983S, CST, USA), phosphorylated-mTOR (phospho-mTOR) (1 : 1000, 5536S, CST, USA), LC3 (1 : 1000, ab48394, Abcam, USA), Beclin1 (1:1000, 3495, CST, USA). The membrane was rinsed three times (5 min/time) with tris buffered saline tween (TBST), incubated with corresponding secondary antibodies at room temperature for 1 h, and washed three times (5 min/time) with TBST again. GAPDH was used as internal reference (1 : 5000, KC-5G5, Shanghai Kangcheng Biological Engineering Co., Ltd., Shanghai, China), and Bio-rad Gel Dol EZ imager (GEL DOC EZ IMAGER, Bio-rad, California, USA) was used for developing. The gray values of protein bands were analyzed using Image J software. Each experiment was repeated three times.

### Cell culture

Human MM cell lines (U266 and MOLP-2), purchased from Shanghai Cell Bank of Chinese Academy of Sciences, were cultured with 10% inactivated fetal bovine serum (FBS) (No. 2494401, Gibco Company, Grand Island, NY, USA) and 1640 culture medium (No. A2494401, Gibco Company, Grand Island, NY, USA) containing 100 units/ml penicillin and 100 mg/ml streptomycin (No. 15140122, Gibco Company, Grand Island, NY, USA) in a 37°C and 5% CO_2_ constant temperature incubator. When cells reached 80% confluence, the cells were digested by 0.25% trypsin and passaged. The drug resistant cell line was established by gradually increasing the concentration of melphalan in MOLP-2 cell culture medium. The initial and the final concentrations of melphalan were 0.25 μmol/L and 5 μmol/L, respectively, with an increase of 0.25 μmol/L every two weeks. The drug resistant cell line was obtained after 40-weeks’ *in vitro* culture. The drug resistant cell was designated as MM MOLP-2/R for later drug resistance experiment.

### Cell grouping

U266 cells were divided into four groups: blank (normally cultured U266 cells), tunicamycin (TM) (2 μg/ml tunicamycin, T776, Sigma Aldrich, USA), TM + insulin-like growth factor-1 (IGF-1) (2 μg/ml tunicamycin and 13 nM IGF-1[Cell Sciences, MA, USA]) and TM + rapamycin (2 μg/ml tunicamycin and 100 nM rapamycin [Calbiochem, San Diego, CA, USA]). IGF-1 is an activator of the PI3K/Akt/mTOR signaling pathway, but rapamycin is an inhibitor of the mTOR signaling pathway. The MM drug resistant cell lines (MOLP-2/R) were divided into four groups: blank (normally cultured MOLP 2/R cells), TM (2 μg/ml tunicamycin), TM + IGF-1 (2 μg/ml tunicamycin and 13 nM IGF-1) and TM + rapamycin (2 μg/ml tunicamycin and 100 nM rapamycin).

### Monodansylcadaverine (MDC) staining

Twenty-four hours after cell treatment, the supernatant was discarded, and the cells were washed twice with phosphate buffered saline (PBS) and incubated in a constant temperature incubator at 37°C with 5% CO_2_ for 20 min in the dark, with 50 nM MDC in each well. A plate with six wells was selected and placed under a fluorescence microscope to observe cell autophagy within 1 h and photographs were taken. Each experiment was repeated three times.

### Flow cytometry

Twenty-four hours after cell treatment, the cells of each group were washed once with 1 × PBS, resuspendeded with PBS containing 75% ethanol and 0.5 mmol/L ethylene diamine tetraacetic acid (EDTA) and then fixed at 4°C for 1 h. The cells were centrifuged at 2000 rpm for 5 min with the supernatant discarded, washed once with 1 × PBS and resuspended with 500 μL of PBS solution containing 0.1% triton x-100 solution (Triton X-100) and 50 μg/mL ribonuclease (RNase). Then 90 μL of staining solution containing 0.5 mg/mL of the third fluorescence channel (FL3) and the first fluorescence channel (FL1) were quickly added and mixed evenly using a pipetting gun, making them react without light at room temperature for 30 min. The cells were filtered with nylon membrane and analyzed by EPICS XL-4 flow cytometer (COULTER, USA). The percentage of FL3^+^/FL1^+^ cells in total cells was considered as the rate of autophagy. Each experiment was repeated three times.

### Annexin V-FITC/propidium iodide (PI) staining

Twenty-four hours after cell treatment, the cells of each group were washed two times with PBS, digested by 0.25% trypsin, and centrifuged at 1000 rpm for 10 min. The cells were collected, washed three times with PBS, resuspended and counted. The concentration of cells was adjusted to 5 × 105 cells/ml, and 5 μL of Annexin V FITC was added into each 100 μL of cells and then gently mixed. The cells were incubated without light at room temperature for 10 min and centrifuged at 1000 rpm for 5 min. The supernatant was discarded and 10 μL of PI staining solution was added and mixed gently with cell suspension liquid. Flow cytometry was used to measure cell apoptosis. The percentage of Annexin V^+^/PI^+^ cells in total cells was recorded as the apoptosis rate. Each experiment was repeated three times.

### Cell count kit-8 (CCK-8) assay

After the cells in each group were treated for 0 d, 1 d, 2 d, and 3 d, CCK-8 reagent was added into 96-well plates (20 μL/well). The cells were removed from the incubation box after 4 h, and a microplate reader was used to detect optical density (OD) value (A) at 490 nm. A proliferation graph was drawn with time as the abscissa and the OD values as the vertical coordinate. Each experiment was repeated three times.

### Statistical analysis

Data were analyzed by SPSS 21.0 statistical software (SPSS Inc., Chicago, IL, USA). The measurement data were expressed as mean ± standard deviation (SD). Normally distributed data from two groups were compared using *t*-test, and the comparison among multiple groups was done using one-way analysis of variance (ANOVA). A value of *P* < 0.05 was considered statistically significant.
